# Small Things Matter: Relevance of MicroRNAs in Cardiovascular Disease

**DOI:** 10.3389/fphys.2020.00793

**Published:** 2020-07-07

**Authors:** Linsey J. F. Peters, Erik A. L. Biessen, Mathias Hohl, Christian Weber, Emiel P. C. van der Vorst, Donato Santovito

**Affiliations:** ^1^Institute for Molecular Cardiovascular Research, RWTH Aachen University, Aachen, Germany; ^2^Department of Pathology, Cardiovascular Research Institute Maastricht, Maastricht University Medical Centre, Maastricht, Netherlands; ^3^Interdisciplinary Center for Clinical Research, RWTH Aachen University, Aachen, Germany; ^4^German Centre for Cardiovascular Research, Partner Site Munich Heart Alliance, Munich, Germany; ^5^Klinik für Innere Medizin III, Universität des Saarlandes, Homburg, Germany; ^6^Institute for Cardiovascular Prevention, Ludwig-Maximilians-University Munich, Munich, Germany; ^7^Department of Biochemistry, Cardiovascular Research Institute Maastricht, Maastricht University Medical Centre, Maastricht, Netherlands; ^8^Munich Cluster for Systems Neurology, Munich, Germany

**Keywords:** microRNAs, cardiovascular diseases, cardiovascular risk factors, atherosclerosis, myocardial infarction, cardiac remodeling, therapy, biomarker

## Abstract

MicroRNAs (miRNAs) are short sequences of non-coding RNA that play an important role in the regulation of gene expression and thereby in many physiological and pathological processes. Furthermore, miRNAs are released in the extracellular space, for example in vesicles, and are detectable in various biological fluids, such as serum, plasma, and urine. Over the last years, it has been shown that miRNAs are crucial in the development of several cardiovascular diseases (CVDs). This review discusses the (patho)physiological implications of miRNAs in CVD, ranging from cardiovascular risk factors (i.e., hypertension, diabetes, dyslipidemia), to atherosclerosis, myocardial infarction, and cardiac remodeling. Moreover, the intriguing possibility of their use as disease-specific diagnostic and prognostic biomarkers for human CVDs will be discussed in detail. Finally, as several approaches have been developed to alter miRNA expression and function (i.e., mimics, antagomirs, and target-site blockers), we will highlight the miRNAs with the most promising therapeutic potential that may represent suitable candidates for therapeutic intervention in future translational studies and ultimately in clinical trials. All in all, this review gives a comprehensive overview of the most relevant miRNAs in CVD and discusses their potential use as biomarkers and even therapeutic targets.

## Introduction

MicroRNAs (miRNAs) are short sequences (∼22 nucleotides) of endogenous non-coding RNA that emerged as a class of negative post-transcriptional regulators of gene expression. Hundreds of different miRNAs have been identified in humans with many of them showing highly conserved sequences and preferential target transcripts across species (Kozomara and Griffiths-Jones, 2014). The post-transcriptional regulation mediated by miRNAs takes place in the RNA-induced silencing complex (RISC), a macromolecular complex where miRNAs loaded into the protein Argonaute-2 (AGO2) interact with the 3′untranslated region (3′UTR) of target RNAs. This interaction allows the recruitment of multiple additional proteins (e.g., TNRC6, PABPC) which favor decay of target RNAs or inhibition of their translation, hence realizing the negative regulation of gene expression (Bartel, 2018). Additionally, some specific miRNAs (e.g., miR-126-5p) have recently been shown to operate also through uncanonical mechanisms which contribute to their ultimate effects on cellular homeostasis and function (Dragomir et al., 2018; Santovito et al., 2020a). As a class, miRNAs are able to regulate the expression of multiple effectors playing crucial roles in developmental processes as well as in human diseases. Interestingly, miRNAs are also released in the extracellular space, for example in vesicles (i.e., exosomes, microparticles, apoptotic bodies), as also reviewed by Davidson et al. (2019), and can be bound to carrier proteins such as AGO2, or associated to plasma lipoproteins (Arroyo et al., 2011; Vickers et al., 2011; Loyer et al., 2014b). Mechanisms for selective sorting and release of miRNAs (especially in exosomes) are under investigation and involves the raft-like regions in vesicular membranes, the sumoylation of RNA-binding proteins, the secretive autophagy and the LC3-conjugation machinery (Villarroya-Beltri et al., 2013; Janas et al., 2015; Leidal et al., 2020). Extracellular miRNAs can convey messages into recipient cells, thus realizing a paracrine (and possibly endocrine) inter-cellular communication system (Valadi et al., 2007; Zernecke et al., 2009; Zhang et al., 2017). Noteworthy, extracellular miRNAs are detectable in human biological fluids such as serum, plasma, urine, and tears with peculiar changes in their expression associated with multiple diseases and regulated by treatments. The stability of circulating miRNAs and their regulation in pathological conditions raised the intriguing possibility of their use as disease and prognostic biomarkers for human diseases (Zampetaki et al., 2012a; Hayes et al., 2014; Santovito and Weber, 2017; Tielking et al., 2019). The relevance of miRNAs in the cardiovascular system was suggested by evidence that deletion of *Dicer*, the rate-limiting enzyme for maturation of miRNAs, in mice resulted in embryonic death due to defective angiogenesis and subsequent hemorrhages (Yang et al., 2005). Starting from this early evidence, multiple studies have confirmed the expression of miRNAs in human cardiac and vascular tissues and provided strong evidence on the role of miRNAs in the development and progression of multiple cardiovascular diseases (CVDs). Here, we will discuss the (patho)physiological implication of miRNAs in cardiovascular risk factors, atherosclerosis, cardiac remodeling, and myocardial infarction. Additionally, miRNAs are of course also being studied in other CVDs, such as myocarditis and arrhythmia. However, this review will not focus on these pathologies, as they have already been extensively reviewed elsewhere (Bátkai and Thum, 2012; Tijsen et al., 2012; Kim, 2013; Santulli et al., 2014; Marques and Charchar, 2015; Hijmans et al., 2018). Moreover, we will review the potential application of miRNAs as biomarker and therapeutic targets. As recent years enormous amounts of studies have focused on miRNAs in the discussed topics, we will only highlight the most relevant miRNAs that have been described in multiple studies or seem highly promising for clinical applications.

## Cardiovascular Risk Factors

**FIGURE 1:**
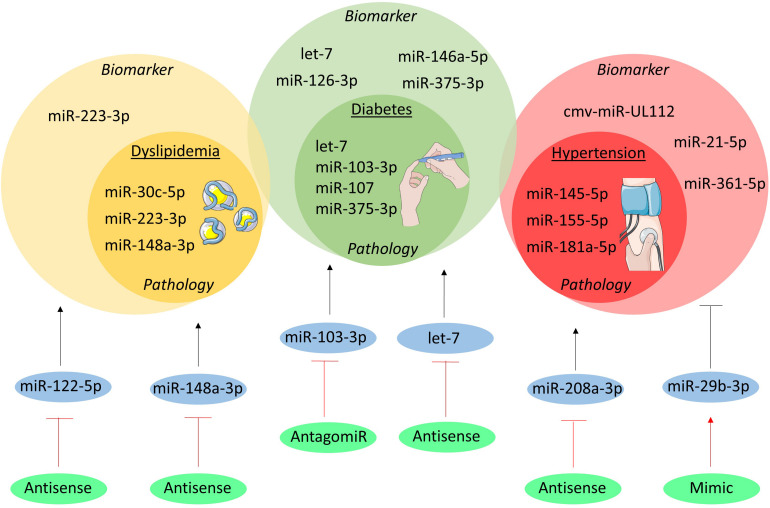
MicroRNAs in cardiovascular risk factors. The miRNAs involved in the pathogenesis of dyslipidemia, diabetes and hypertension are shown in the inner yellow, green, and red circles, respectively. In the outer circles the miRNAs are listed which have been studied as biomarker. Moreover, the therapeutically targeted miRNAs are shown in blue circles and their initial effect on the cardiovascular risk factors is depicted as a black, stimulatory or inhibitory arrow. The green circles indicate the method of intervention and their effect on the miRNA is visualized as a red, stimulatory or inhibitory arrow.

Clinical and pre-clinical studies highlight the influence of miRNAs in the complex pathways underlying medical conditions (i.e., dyslipidemia, diabetes, hypertension) which increase the risk of cardiovascular events (Figure 1).

### Pathology

Genome-wide association studies revealed an intriguing association between abnormalities of plasma lipids and 69 miRNAs that regulate the expression of key genes in lipoprotein metabolism (Wagschal et al., 2015). Among them, miR-148a-3p is predominantly expressed in the liver and controls, at post-transcriptional level, the expression and function of low-density lipoprotein (LDL)-receptor and ATP-binding cassette transporter A1 (ABCA1) (Goedeke et al., 2015). Similarly, miR-223-3p regulates both cholesterol synthesis via the repression of 3-hydroxy-3-methylglutaryl-CoA synthase 1 (HMGCS1) and methylsterol mono-oxygenase 1 (SC4MOL) as well as cholesterol efflux through regulation of scavenger receptor class B type 1 (SR-B1) and ABCA1 (Vickers et al., 2014). In addition, miR-30c-5p affects *de novo* lipid biosynthesis in the liver and the release of ApoB-containing lipoproteins, probably through the regulation of expression and function of lysophosphatidylglycerol acyltransferase 1 (LPGAT1) and microsomal triglyceride transfer protein (MTTP), respectively (Soh et al., 2013). Finally, strong evidence on the regulatory role of miRNAs in reverse cholesterol transport and high-density lipoproteins (HDL) metabolism is accumulating, as extensively reviewed elsewhere (Canfran-Duque et al., 2016).

Besides lipid metabolism, miRNAs also regulate glucose metabolism and are thereby involved in the pathophysiology of diabetes. Conditional Dicer knock-out in the pancreas (*Pdx^*Cre*^Dicer^*fl/fl*^*) impedes the formation of Langerhans islets and differentiation of insulin-producing β-cells (Lynn et al., 2007). Highly expressed in the β-cell, miR-375-3p controls insulin synthesis under hyperglycemia conditions and prevents its release (Poy et al., 2004; El Ouaamari et al., 2008). Interestingly, higher miR-375-3p expression is observed in β-cells of patients with type 2 diabetes, thereby suggesting its relevance in this human disease (Zhao et al., 2010). Insulin sensitivity is also regulated by miRNAs as for example the let-7 miRNA-family participates in pathways leading to impaired insulin sensitivity in the skeletal muscle. This effect is mediated by targeting insulin receptor substrate (IRS)-2 and insulin-like growth factor (IGF)-1 receptor and antagonizing let-7 by RNA-interference or transgenic overexpression of two negative modulators of let-7 biogenesis (Lin28a and Lin28b) improved insulin sensitivity and glucose homeostasis (Frost and Olson, 2011; Zhu et al., 2011). On the other hand, the miR-103/-107 family is up-regulated in the liver of obese mice and governs hepatic insulin sensitivity. Indeed, overexpression of miR-107 increases hepatic gluconeogenesis, resulting in hyperinsulinemia and hyper-glycemia, while treatment with an antagomir against miR-103-3p improves glycemic tolerance, insulin sensitivity, and decreases both subcutaneous and visceral adipose tissue in mice (Trajkovski et al., 2011).

Finally, miRNAs participate in the regulation of blood pressure and development of hypertension by affecting the renin-angiotensin axis. Indeed, a single-nucleotide-polymorphism (rs5186) disrupts the binding site for miR-155-5p in the type 1 angiotensin II receptor (AGTR1) mRNA and is associated with increased risk for hypertension (Bonnardeaux et al., 1994; Sethupathy et al., 2007). The expression of AGTR1 is higher in young hypertensive patients carrying the mutant allele, directly correlated with arterial blood pressure, and inversely correlated with miR-155-5p expression in blood mononuclear cells (Ceolotto et al., 2011). Being hosted on chromosome 21, miR-155-5p could also contribute to the lower blood pressure in patients with Down syndrome (Sethupathy et al., 2007). Additionally, miR-181a-5p regulates the expression of renin *in vitro*, it is downregulated in a murine model of neurogenic hypertension and in the renal cortex of hypertensive patients, and inversely correlated with higher renin mRNA expression (Marques et al., 2011; Jackson et al., 2013). Finally, miR-145-5p regulates angiotensin-converting enzyme (ACE) in vascular smooth muscle cells (VSMCs) and *in vivo* deficiency results in hypotension and impaired vascular reactivity (Boettger et al., 2009). Additionally, changes in miR-145 expression were found in hypertensive patients in blood mononuclear cells and in carotid atherosclerotic plaques (Santovito et al., 2013; Kontaraki et al., 2014). Overall, the role of miRNAs in the regulation of lipid and glucose metabolism and vascular reactivity has been associated with important implications for the development of pathological abnormalities, like hypertension, diabetes and atherosclerosis.

### Biomarker

Dyslipidemia and diabetes are diagnosed by routine blood tests. Nonetheless, alterations identified in circulating miRNAs could contribute to a better characterization of the patients and unveil new molecular pathways. For examples, miR-223-3p does not only control hepatic cholesterol and lipoproteins metabolism, but is also loaded into HDL to be released in conditions of hypercholesterolemia as shown in patients with familial hypercholesterolemia (Vickers et al., 2011). However, diabetes is among the most investigated diseases in regards of circulating miRNAs. Among the miRNAs affected in their circulating levels, there are those involved in the above-mentioned mechanisms of insulin release and sensitivity (i.e., miR-375-3p, the let-7 family), those involved in inflammation (e.g., miR-146a-5p), and those influencing endothelial biology such as miR-126-3p (Zampetaki et al., 2010; Kong et al., 2011; Santovito et al., 2014). This finding further supports the existence of profound crosstalk between diabetes, endothelial (dys)function, and atherosclerosis. Finally, anti-diabetic treatment could promote a (partial) reversion of the abnormalities in circulating miRNAs, hence highlighting their possible use to monitor therapeutic efficacy (Ortega et al., 2014; Santovito et al., 2014).

Hypertension has been associated with anomalies of circulating miRNAs. Although not essential for diagnostic purposes, circulating miRNAs may own additive prognostic value. For example, whole blood expression of miR-361-5p could discriminate patients affected by salt-sensitive vs. salt-resistant hypertension, thus possibly supporting the clinical decision of the appropriate anti-hypertensive treatment (Qi et al., 2017). Moreover, higher plasma levels of miR-21-5p positively correlated with carotid intima-media thickness in hypertensive patients, nominating this miRNA as a non-invasive marker of carotid atherosclerosis in these patients (Cengiz et al., 2015). Finally, an intriguing study showed an increase of circulating cmv-miR-UL112, a miRNA encoded by the human cytomegalovirus, in patients with hypertension (together with changes of endogenous miR-296-5p and let-7e-5p), thus proposing a link between cytomegalovirus infection and hypertension that mandate additional experimental proof (Li et al., 2011).

### Therapeutic Potential

Therapeutic modulation of miRNAs affects serum lipoproteins in experimental studies. A remarkable evidence is provided by a clinical trial employing anti-sense interference of miR-122-5p (known as Miravirsen). Although this was a phase 2 clinical trial investigating safety and efficacy of miR-122-5p inhibition for Hepatitis C, treatment resulted in a dose-dependent decrease of serum total cholesterol levels (Janssen et al., 2013). The underlying mechanisms are not yet fully elucidated, although experiments in animal models identified 3-hydroxy-3-methylglutaryl-CoA reductase (HMGCR), the key enzyme for intracellular cholesterol biosynthesis, and MTTP, which transfers triglycerides onto ApoB during very low-density lipoprotein (VLDL) biogenesis, as two relevant targets (Esau et al., 2006; Tsai et al., 2012). Therapeutic modulation of other miRNAs to improve lipid and glucose metabolism has also been tested in animal models. For example, inhibition of miR-148a-3p by antisense nucleotides resulted in lower LDL cholesterol and higher HDL cholesterol in serum, reflecting the upregulation of hepatic LDL-receptor and ABCA1 (Goedeke et al., 2015). Moreover, inhibition of miR-103-3p by antagomir resulted in lower plasma glucose and improved insulin resistance in liver and adipose tissue of obese mice (Trajkovski et al., 2011). Similarly, systemic administration of an antisense nucleotide targeting the whole let-7 family prevented impaired glucose tolerance in obese mice by improving hepatic and muscular insulin sensitivity (Frost and Olson, 2011). These data designate miR-103-3p and let-7 as possible therapeutic targets in diabetes.

Finally, miRNAs have been experimentally tested as therapeutic agents in hypertension and related organ damages. Systemic administration of antisense nucleotides against miR-208a-3p blunted cardiac stress and consequent pathological hypertrophy in hypertensive rats (Dickinson et al., 2013). Moreover, treatment with miR-29b-3p after Angiotensin II-induced hypertension improved the progressive impairment of cardiac function and reverted histological markers of hypertensive cardiopathy in mice (Zhang et al., 2014). These studies unveil the therapeutic potential of miRNAs in hypertension and its complications.

## Atherosclerosis

Atherosclerosis is the main underlying cause of many CVDs. It is a chronic inflammatory disease of the arterial walls that eventually determine vessel stenosis and acute occlusion by atherothrombosis. The pathophysiological mechanisms leading to atherosclerosis progression are numerous and complex as reviewed elsewhere (Weber and Noels, 2011).

### Pathology

#### Endothelial Cells

**FIGURE 2:**
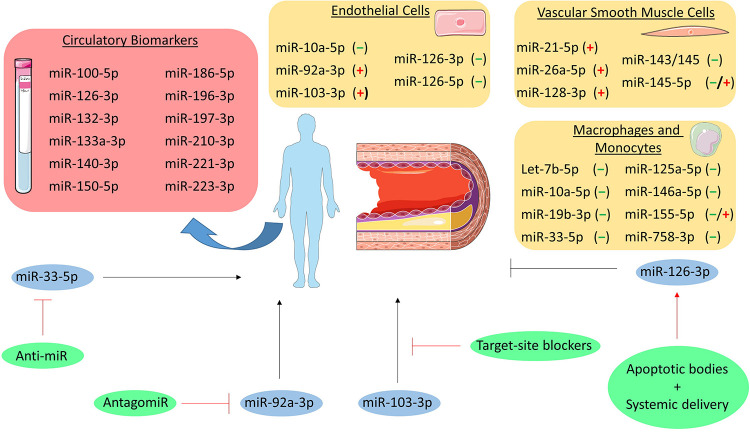
MicroRNAs in atherosclerosis. MiRNAs involved in the pathogenesis of atherosclerosis are grouped per cell type (i.e., endothelial cells, vascular smooth muscle cells, and macrophages and monocytes). The (–), (+), and (–/+) indicate that the miRNA is either atheroprotective, pro-atherogenic or both, respectively. Additionally, circulatory biomarker miRNAs are listed in the red box. Furthermore, the miRNAs that have been studied as therapeutic target are depicted in blue circles and their initial effect on atherosclerosis is shown as a black, stimulatory or inhibitory arrow. The therapeutic intervention is shown in green circles and the effect on the miRNA is depicted as a red, stimulatory or inhibitory arrow.

Several lines of evidence show the involvement of miRNAs in all stages of atherosclerosis (Figure 2). Amongst the most expressed miRNAs in endothelial cells (ECs), especially the miR-126 duplex plays a crucial role in regulating endothelial function. In contrast to most of the miRNAs, the precursor of miR-126 gives rise to two stable mature miRNA strands (miR-126-3p and miR-126-5p) which both coordinate atheroprotective functions. Indeed, miR-126-3p can regulate angiogenesis and reduces inflammatory leukocyte adhesion to ECs by repressing targets such as the vascular cell adhesion molecule-1 (VCAM-1) (Fish et al., 2008; Harris et al., 2008). On the other hand, miR-126-5p not only promotes the proliferative capacity by targeting delta-like 1 (Dlk1), a negative regulator of the NOTCH1 pathway (Schober et al., 2014), but also protects ECs from apoptotic cell death through an uncanonical mechanism. Indeed, high shear stress promotes nuclear localization of miR-126-5p by a pathway involving the activation of autophagy and the RNA-binding protein Mex3a (Dragomir et al., 2018; Santovito et al., 2020a). Nuclear miR-126-5p acts as an aptamer by directly binding to the effector caspase-3, inhibiting its catalytical activity, and protecting ECs from apoptosis (Santovito et al., 2020a). Besides the intracellular role, the guide strand of miR-126 (miR-126-3p) is released via apoptotic bodies and mediates a paracrine signaling regulating CXCL12 release (Zernecke et al., 2009). The relevance of the cell-specific contribution of CXCL12 and its receptor CXCR4 in the progression of atherosclerosis has been extensively proven (van der Vorst et al., 2015; Doring et al., 2017, 2019), thus further linking the miR-126 duplex to atherosclerosis development.

Besides the miR-126 duplex, other EC-related miRNAs have been demonstrated to play a role in atherosclerosis. Studies involving a murine model of conditional deletion of *Dicer* in ECs (*Cdh5^*Cre*^Dicer^*fl/fl*^*) revealed a detrimental role for miR-103-3p in atherosclerosis. Indeed, miR-103-3p promotes endothelial inflammation by targeting Kruppel-like factor 4 (KLF4) and inhibits EC proliferation and promotes DNA damage through targeting of lncWDR59 in areas of disturbed shear stress (Hartmann et al., 2016; Natarelli et al., 2018). Silencing of *Dicer* also associated with higher expression of KLF2, an effect mediated by miR-92a-3p (Wu et al., 2011), a miRNA upregulated in atheroprone vascular areas, to promote endothelial activation and atherosclerosis progression (Loyer et al., 2014a). Furthermore, miR-92a-3p can be released in extracellular vesicles, thereby driving an atheroprone phenotype in recipient macrophages by targeting KLF4 (Chang et al., 2019) and regulating ECs angiogenic ability through a thrombospondin 1 (THBS1)-dependent mechanism (Liu et al., 2019). In contrast, miR-10a-5p is downregulated in atherosusceptible areas to foster IκB/NF-κB-mediated infiammation by repressing MAP3K7 and β-TRC (Fang et al., 2010).

#### Smooth Muscle Cells

Besides their role in ECs, miRNAs expressed in VSMCs also contribute to atherosclerosis and arterial remodeling. As one of the most enriched, the miR-143/-145 cluster acts as a phenotypical regulator of VSMCs. These miRNAs prevent proliferation and acquisition of a pro-atherosclerotic synthetic phenotype, with the effects being mediated by multiple targets such as KLF4, KLF5, and ACE (Boettger et al., 2009; Cheng et al., 2009; Cordes et al., 2009). Interestingly, this miRNA cluster is involved in an atheroprotective intercellular crosstalk between VSMCs and ECs. While miR-143/-145 produced in ECs favors the acquisition of an atheroprotective phenotype of VSMCs (reduced proliferation and dedifferentiation) upon vesicle-mediated transfer (Hergenreider et al., 2012), miR-143/-145 is also transferred from VSMCs to nearby ECs via tunneling nanotubes and contributes to stabilization of the endothelium (Hergenreider et al., 2012; Climent et al., 2015). Finally, a circular RNA (namely *circ_Lrp6*) acts as a natural sponge for miR-145-5p dampening its activity (Hall et al., 2019), highlighting a further regulatory layer for miR-145-5p activities. However, the ultimate relevance of miR-145-5p in atherosclerosis requires additional investigations as beneficial effects were observed by local vascular overexpression (Lovren et al., 2012; Hall et al., 2019) as well as by genetic deletion (Xin et al., 2009; Sala et al., 2014). Other miRNAs also contribute to VSMCs phenotype: miR-128-3p regulates methylation of the *Myh11* gene (crucial for the contractile phenotype) by targeting KLF4, thus preventing dedifferentiation (Farina et al., 2020); miR-21-5p promotes VSMCs proliferation and neointima formation thought repressing phosphatase and tensin homolog (PTEN) expression (Ji et al., 2007); miR-26a-5p facilitates VSMCs proliferation and migration affecting the TGF-β pathway (Leeper et al., 2011).

#### Monocytes and Macrophages

Monocytes and macrophages also express miRNAs that are involved in atherosclerosis development. Genetic deletion of *Dicer* in macrophages (*LysM^*Cre*^Dicer^*fl/fl*^*) exacerbated atherosclerosis by enhancing inflammatory activation and favoring formation of foam cells due to an impaired mitochondrial fatty acid metabolism (Wei et al., 2018). The metabolic effects were reversed by re-expression of miR-10a-5p and let-7b-5p with consequent repression of *Lcor* (Wei et al., 2018). miR-155-5p, which is enriched in hematopoietic cells, is involved in atherosclerosis featuring opposite roles during early and advanced stages of the disease. Its expression in macrophages increases during atherogenesis and, while it suppresses early lesion formation by inhibiting macrophage proliferation, it promotes inflammatory activation and reduces efferocytosis at advanced stages (Donners et al., 2012; Nazari-Jahantigh et al., 2012; Du et al., 2014; Wei et al., 2015). Moreover, miR-146a-5p induced by inflammatory stimuli participates to resolution of inflammation by restraining inflammatory cytokines expression, reducing oxidized LDL (oxLDL) uptake, and protecting against atherosclerosis (Taganov et al., 2006).

Macrophage cholesterol uptake and efflux is also regulated by miRNAs. The uptake of oxidized lipoprotein is inhibited *in vitro* by miR-125a-5p and miR-155-5p by down-regulating the scavenger receptors CD68 and LOX1 (Chen et al., 2009; Huang et al., 2010). However, the cholesterol efflux pathways are the most affected by miRNA regulation. Cholesterol efflux first requires conversion of cholesteryl esters (stored in lipid droplets) into free cholesterol mediated by neutral cholesteryl ester hydroxylases or by autophagy. This latter process is repressed by miR-155-5p in macrophages and could contribute to reduced cholesterol efflux upon lipid (over)loading (Du et al., 2014). Free cholesterol is then transferred to apolipoprotein A1 or mature HDL by the synergic action of ABCA1 and ABCG1, respectively. In macrophages, these transporters are strongly regulated by multiple miRNAs such as miR-33-5p, miR-758-3p, and miR-19b-3p (Najafi-Shoushtari et al., 2010; Rayner et al., 2010; Ramirez et al., 2011; Lv et al., 2014). Interestingly, inflammatory stimuli (e.g., lipopolysaccharide) upregulate the expression of miR-33-5p in macrophages which stimulates an inflammatory phenotype (Ouimet et al., 2015), suggesting an additional link between lipid homeostasis and inflammation status. Unlike mice, humans express two miR-33 homologs (miR-33a-5p and miR-33b-5p) that exhibit a different regulation. Indeed, while miR-33a-5p is not affected (or slightly downregulated), miR-33b-5p is upregulated in atherosclerotic plaques from hypercholesterolemic patients and is paralleled by a lower translation rate of the ABCA1 protein (Mandolini et al., 2015). The presence of two miR-33 homologs should be considered while translating murine findings into a human disease.

### Biomarker

Atherosclerotic plaque disruption underlies the development of acute ischemic syndromes and identification of vulnerable plaques is an unmet need in medical research. Studies performed on human unstable atherosclerotic plaques revealed peculiar changes in the expression profiles of miRNAs, including miR-100-5p, miR-127-3p, miR-133a-3p, miR-210-3p, miR-221-3p (Cipollone et al., 2011; Maitrias et al., 2015; Eken et al., 2017). These studies allowed the investigation of regulated targets and pathways influencing the mechanisms of plaque destabilization, however miRNAs analysis in atherosclerotic lesions may have limited application in clinical practice. Interestingly, the circulating levels of some of these miRNAs (i.e., miR-100-5p, miR-133a-3p, miR-210-3p, miR-221-3p) are also altered in patients with vulnerable carotid or coronary atherosclerotic plaques (Tsai et al., 2013; Wang et al., 2013; Soeki et al., 2015; Eken et al., 2017), suggesting their role as possible biomarkers to identify patients with vulnerable plaques. However, independent cohorts should be analyzed with standardized analytical procedures to properly validate the findings, assess diagnostic performance, and prove clinical utility.

Circulating miRNAs have also been tested for their ability to predict cardiovascular events. As one of the miRNAs regulated in vulnerable plaques (Cipollone et al., 2011; Maitrias et al., 2015), high plasma miR-133a-3p levels were associated with a higher risk of cardiovascular events in a prospective study in patients with familiar hypercholesterolemia with a follow-up of 8 years (Escate et al., 2020). Furthermore, a prospective population-based survey unveiled the association of miR-126-3p, miR-197-3p, and miR-223-3p with the incidence of myocardial infarction over a 10-year follow-up period (Zampetaki et al., 2012b). Finally, higher miR-19b-3p, miR-132-3p, miR-140-3p, miR-150-5p, miR-186-5p levels were linked to a high degree of cardiovascular deaths in the following 4 years in patients with coronary artery disease (Karakas et al., 2017).

#### Therapeutic Potential

Systemic and local administration of mimics, antagomirs, and target-site blockers was employed in animal models to explore the therapeutic potential of miRNA modulation *in vivo*. For example, delivery of miR-126-3p via apoptotic bodies as well as systemic treatment with the miR-126-5p mimic reduced atherosclerotic lesion formation in mice (Zernecke et al., 2009; Schober et al., 2014), hence supporting the beneficial role of the miR-126 duplex in vascular homeostasis. On the other hand, beneficial effects were observed by the inhibition of endothelial miRNAs with detrimental functions. Indeed, inhibition of miR-92a-3p by injection of an antagomir (Loyer et al., 2014a) or disruption of the interactions between miR-103-3p and its proatherogenic targets (Hartmann et al., 2016; Natarelli et al., 2018) reduced atherosclerosis and improved the lesion phenotype.

The inhibition of miR-33-5p was also extensively investigated, as cholesterol efflux and reverse transport represent an intriguing therapeutic opportunity for atherosclerosis regression. In mice, short-term (4 weeks) anti-miR-33 treatment increased cholesterol efflux from lesional macrophages and promotes regression of atherosclerosis (Rayner et al., 2011). However, long-term treatment (14 weeks) was not associated with beneficial effects on atherosclerosis (Marquart et al., 2013). A possible explanation is the concomitant hepatic overexpression of genes regulating fatty acid synthesis (e.g., acetyl-CoA carboxylase) with consequent increased plasma triglyceride levels and liver steatosis (Goedeke et al., 2014). Notably, long-term treatment of non-human primates, which express both miR-33 homologs, did not cause liver toxicity (Rottiers et al., 2013). It is therefore possible that the lack of miR-33b in rodents does not allow the complete evaluation of underlying mechanisms in murine models. In example, lower miR-33b-5p expression in human atherosclerotic plaques is observed after treatment with rosuvastatin (Santovito et al., 2020b), possibly contributing to anti-atherosclerotic properties of this molecule. Therefore, miR-33 represents a promising target for atherosclerosis, although further investigations are required to verify safety and efficacy in primates.

## Myocardial Infarction

Myocardial infarction (MI) is characterized by the necrosis of myocytes followed by extracellular matrix (ECM) deposition by activated cardiac fibroblasts and consequent scar formation. This pathology is defined as cardiomyocyte death due to prolonged ischemia mostly caused by coronary artery disease.

## Pathology

**FIGURE 3:**
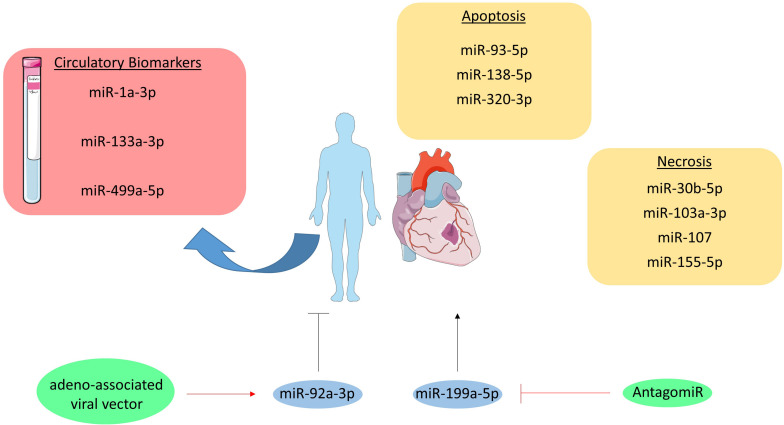
MicroRNAs in myocardial infarction. MiRNAs involved in the pathogenesis of myocardial infarction are grouped per cell death type (i.e., apoptosis and necrosis). Moreover, the circulatory miRNAs studies as biomarker are listed in the red box. Furthermore, the therapeutically targeted miRNAs are visualized in blue circles and their initial effect on myocardial infarction is shown as a black, stimulatory or inhibitory arrow. The therapeutic intervention is depicted in green circles and their effect on the targeted miRNA is shown as a red, stimulatory or inhibitory arrow.

Myocardial infarction usually involves different types of cell death including necrosis and apoptosis, which are regulated amongst others by miRNAs (Figure 3; Higashi et al., 2015; Wang J.X. et al., 2015). Both pro- and anti-apoptotic miRNAs have been identified regarding apoptosis, such as miR-93-5p, miR-138-5p, and miR-320-3p. miR-93-5p was found to be anti-apoptotic in cardiomyocytes by targeting PTEN in mice with ischemia/reperfusion (I/R) injury (Ke et al., 2016). Additionally, in an *in vitro* study miR-138-5p protects cardiac myoblasts against hypoxia-induced apoptosis via the MLK3/JNK/c-jun pathway (He et al., 2013). In contrast, miR-320-3p targets IGF-1 and thereby promotes apoptosis in cardiomyocytes (Song et al., 2016).

Additionally, miRNAs like miR-103a-3p, miR-107, miR-155-5p, and miR-30b-5p are either anti- or pro-necrotic in cardiac disorders (Liu et al., 2011; Wang K. et al., 2015,b). For example, miR-103a-3p and miR-107 induce necrosis in cardiomyocytes by targeting Fas-associated protein with death domain (FADD) (Wang J.X. et al., 2015). In contrast, miR-30b-5p inhibits necrosis through targeting cyclophilin D, which usually promotes necrosis. Interestingly, enhanced expression of miR-30b-5p in the heart reduced necrosis and MI size following I/R injury (Wang K. et al., 2015). Likewise, overexpression of miR-155-5p in cardiomyocyte progenitor cells attenuates necrosis by targeting receptor interacting protein 1 (RIP1) (Liu et al., 2011).

### Biomarker

In MI, cardiac troponins are used as the golden standard diagnostic biomarkers. Although the measurement of troponin levels is a sensitive test, it is not specific for MI as for example myocarditis can also alter troponin levels (Halushka et al., 2019). Therefore, the opportunity for miRNAs as biomarker lies in its potential to discriminate true MI from other diseases, which have similar classical biomarker profiles as MI. A number of miRNAs have been explored in the context of MI, such as miR-1a-3p, miR-133a-3p, and miR-499a-5p. miR-1a-3p, miR-133a-3p, and miR-499a-5p levels were all elevated in MI patients, but were not more specific as compared to troponin (Ai et al., 2010; Wang et al., 2010). In contrast, it was found that 93% of 510 patients suffering from MI were tested positive for miR-499a-5p, while only 88% tested positive for troponin (Devaux et al., 2012). Therefore, it seems that miRNAs still have some additional value on top of troponins.

### Therapeutic Potential

The inability of cardiomyocytes to replicate and regenerate the lost contractile tissue raises the need for novel therapies, as the current ones fail to restore this replication-potential. Interestingly, one very recent study investigated the therapeutic potential of miR-199a-5p in pigs. This miRNA was administered in pigs through an adeno-associated viral vector directly after MI was induced, which resulted in improved contractility, increased muscle mass and reduced scar formation one month after MI and miR-199a-5p delivery. However, despite these improvements a longer, uncontrolled expression of this miRNA resulted in sudden death of 7 out of 10 pigs indicating that the dosage of this potential treatment has to be tightly controlled (Gabisonia et al., 2019). Furthermore, miR-92a-3p inhibition on MI recovery in mice was investigated. The mice were injected with an antagomir at 0, 2, 4, 7, and 9 days after MI was induced, after which cardiac function was determined at day 14. Improved heart function and reduced infarction size could be observed in antagomir injected mice compared to the control injected mice (Bonauer et al., 2009).

## Cardiac Remodeling

Cardiac remodeling is generally defined as molecular and cellular changes clinically manifested as changes in heart shape, size and function caused by cardiac load or injury (Cohn et al., 2000). Cardiac hypertrophy and fibrosis are part of this pathology. In cardiac hypertrophy, myocyte death increases the contractile load on neighboring myocytes, which leads to increased myocyte size. As a maladaptive response to the impaired heart performance, cardiac myofibroblasts deposit an excessive amount of excessive ECM in the interstitium, thereby further enhancing cardiac stiffness and dysfunction (Westermann et al., 2011).

### Pathology

**FIGURE 4:**
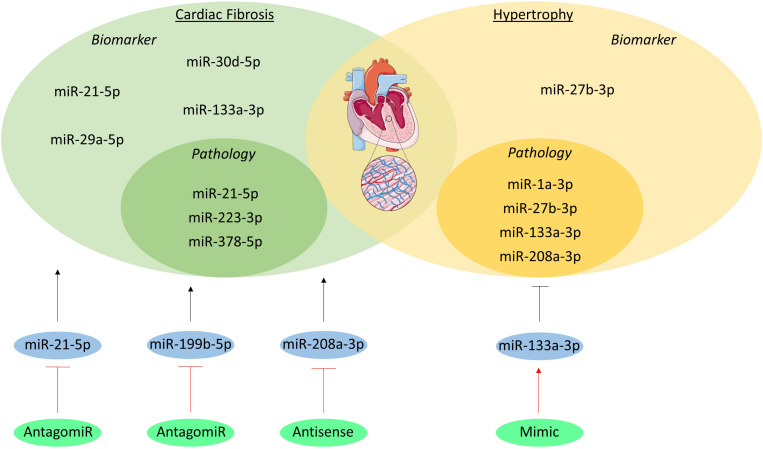
MicroRNAs in cardiac remodeling. The miRNAs involved in the pathogenesis of cardiac fibrosis and hypertrophy are visualized in the inner green and yellow circles, respectively. The miRNAs which have been studied as biomarker are listed in the outer circles. Additionally, the miRNAs studied as therapeutic are shown in blue circles and their initial effect on the pathologies is visualized as a black, stimulatory or inhibitory arrow. The method of intervention is depicted in the green circles and their effect on the miRNA target is shown as a red, stimulatory or inhibitory arrow.

miR-133a-3p, miR-1a-3p, miR-27b-3p, and miR-208a-3p are one of the many miRNAs involved in hypertrophy (Figure 4). miR-133a-3p levels in cardiomyocytes significantly decrease in animal models of hypertrophy and in patients with hypertrophic cardiomyopathy as compared to healthy controls. Moreover, overexpression of this miRNA resulted in preserved cardiac function, whilst inhibition resulted in increased hypertrophy, further associating and even causally linking miR-133a-3p to hypertrophy (Carè et al., 2007). Just like miR-133a-3p, miR-1a-3p has a high abundance in cardiomyocytes and has a lower expression in heart failure patients (Elia et al., 2009). More interestingly, restoration of miR-1a-3p gene expression seems to reverse pressure-induced cardiac hypertrophy in rats (Karakikes et al., 2013). miR-27b-3p has also been shown to be involved in hypertrophy development. *In vivo* silencing of this miRNA with an antagomiR resulted in attenuation of cardiac hypertrophy and dysfunction in mice with heart failure, suggesting that miR-27b-3p promotes cardiac hypertrophy and dysfunction (Wang et al., 2012). miR-208a-3p is also expressed in high levels in healthy cardiomyocytes, where it regulates the balance between the two myosin heavy chains (MHC) isotypes, i.e., α- and β-MHC. This miRNA induces a shift towards the β-MHC isotype, which reduces contractility and is known to be a maladaptive response to cardiac stress (Krenz and Robbins, 2004; Callis et al., 2009).

Several miRNAs have been studied regarding cardiac fibrosis, including miR-21-5p, miR-378-5p, and miR-223-3p. miR-21-5p levels are elevated in cardiac fibroblasts of fibrotic mouse hearts and in heart failure patients (van Rooij et al., 2006). This miRNA also stimulates ECM deposition in mice with increased afterload and myocardial ischemia, which are some of the underlying causes of cardiac remodeling (Thum et al., 2008; Roy et al., 2009). Furthermore, miR-378-5p has been shown to be secreted by cardiomyocytes as a consequence of mechanical stress and it inhibits excessive cardiac fibrosis in an extracellular vesicles-dependent secretory manner (Yuan et al., 2018). Lastly, overexpression of miR-223-3p leads to increased proliferation, differentiation and migration of cardiac fibroblasts and *in vivo* inhibition leads to alleviated cardiac fibrosis (Liu et al., 2018).

### Biomarker

MiRNAs might serve as new serological biomarkers for a precise detection of cardiac remodeling. miR-27b-3p serum levels were measured through stem-loop RT-PCR in 200 hypertensive patients with left ventricular hypertrophy (LVH), 100 hypertensive patients without LVH, and 100 healthy volunteers. Results showed that miR-27b-3p serum levels were significantly higher in the patients with LVH compared to both the hypertensive and healthy subjects without LVH (Wang et al., 2017).

Although cardiac tissue biopsy analysis has been the golden standard for the diagnosis of cardiac fibrosis, circulating biomarkers have been studied to develop a more non-invasive approach (Richards, 2017). Biomarker potential of miRNAs have been well established in myocardial fibrosis, including miR-21-5p, miR-29a-5p, miR-30d-5p, and miR-133a-3p. These were measured in the plasma of left ventricular non-compaction (LVNC) patients both with and without myocardial fibrosis diagnosed with cardiac magnetic resonance with late gadolinium enhancement (LGE), where LGE positivity reflects the presence of fibrosis. Four miRNAs were significantly upregulated in LVNC patient with fibrosis as compared to LVNC patients without fibrosis, suggesting that these miRNAs could be used as biomarkers for detecting fibrosis in the clinic (Szemraj-Rogucka et al., 2019).

### Therapeutic Potential

Cardiac remodeling evokes downregulation of specific miRNAs in the heart; overexpression of these miRNAs is sufficient to induce hypertrophy (van Rooij et al., 2006), creating the opportunity to identify novel targets for miRNA-based therapies. A first *in vivo* study regarding miRNA-based therapies implanted osmotic minipumps in mice for a continues delivery of an antagomir targeting miR-133a-3p. This inhibition of miR-133a-3p resulted in marked and sustained hypertrophy compared to saline infused mice, suggesting an artificial overexpression of miR-133a-3p might have therapeutic value for hypertrophy (Carè et al., 2007). Another study used an antagomir targeting miR-21-5p into mice that underwent a transverse aortic constriction (TAC) operation to induce pressure overload of the left ventricle or into sham operated mice. Silencing miR-21-5p in TAC operated mice resulted in reduced interstitial fibrosis in the heart and attenuated cardiac dysfunction as compared to their controls (Thum et al., 2008). Moreover, *in vivo* inhibition of miR-199b-5p in mice and miR-208a-3p in rats inhibited cardiac remodeling and improved heart function (da Costa Martins et al., 2010; Montgomery Rusty et al., 2011).

## The Road Toward Clinical Application: Current Limitations and Future Challenges

As described in the previous chapters, circulating miRNAs hold promising diagnostic and prognostic potential in CVDs. Yet, further work is necessary to address some aspects that currently limit their use in clinical routine. A large core of evidence derives from monocentric case-control studies, and external validation in large prospective cohorts is often missing. Moreover, anomalies of circulating miRNAs also associate with several non-CVDs (e.g., cancer, inflammatory diseases) as well as concomitant treatments (Hayes et al., 2014; Tielking et al., 2019). However, it is particularly complex to take all these variables into account in studies on circulating miRNAs and appropriate multivariate analyses are sometimes missing or inconclusive, especially in small cohorts. Importantly, there is a relevant lack of standardization in methods and analytical workflows. The most commonly used technique for measuring circulating miRNAs is qPCR with its inherent limitations raised by the lack of unequivocally accepted normalization strategies. Several approaches have been pursued ranging from the use of a synthetic spike-in (e.g., cel-miR-39), to the identification of sets of endogenous miRNAs with the lowest variance among samples, to applying an average of cycle thresholds (Zampetaki et al., 2012a; Halushka et al., 2019). Other techniques have also been applied such as droplet digital PCR, chip-based digital PCR, and RNA-sequencing. However, the disparities in methods and analytical workflows make it difficult to merge results from multiple studies (e.g., meta-analyses). Therefore, all these aspects warrant an urgent need for a consensus. This represents a keystone for proceeding with validation of diagnostic and prognostic performances in multicentric prospective cohorts with the final aim to establish their role in improving risk stratification of patients with CVDs.

Finally, in the light of their pathophysiological relevance, it was a logical consequence to imagine miRNAs as novel therapeutic entities for clinical application. To date, clinical trials (phase 1 and 2) provided proof-of-principle evidence that modulation of miRNAs is feasible in humans (e.g., miravirsen, a miR-122-5p inhibitor) (Janssen et al., 2013). On the other hand, the premature halt of some trials on other miRNA-based therapeutics (i.e., miR-34 mimics) reflects the existence of limiting aspects to overcome. In particular, a single miRNA could target multiple genes and those genes are expressed and regulated in a cell- and tissue-dependent manner, thus evolving the notion of “off-target effects” to an unprecedented complexity, compared to standard drugs. Therefore, tissue-specific delivery is crucial to minimize side effects due to off-targeting of transcripts in other cells types (as in the case of anti-miR-33, see section “Therapeutic Potential”). Although systemic administration can easily reach therapeutic concentrations of miRNAs in organs such as liver and kidney, the delivery in other tissues (e.g., arteries) requires carefully engineered carriers. They include nanoparticles coated with appropriate antigens recognized by receptors unambiguously expressed on targeted cells. However, nanoparticles display low efficiencies in cell internalization and in the release of miRNAs inhibitors in the cytoplasm (1–2%) (Gilleron et al., 2013) thus fostering research to develop new and more effective strategies. In this context, bacterially derived minicells coupled with antibodies against cell-specific markers has been effectively employed for tissue-specific delivery of miR-16 mimics in patients with mesothelioma (van Zandwijk et al., 2017) and may extend the portfolio of cell-specific delivery systems. Another relevant aspect is the influence of chemical modifications of nucleotides on the efficacy and safety of the miRNA-based compounds. Indeed, specific chemistries (e.g., 2′O-methyl nucleotides, Locked Nucleic Acids) are applied to anti-miRNA oligonucleotides to enhance the resistance to serum nucleases, the affinity to their targets, and the pharmacokinetic profile. However, some modifications might influence both the strength of miRNA inhibition and the efficacy of cellular uptake. Moreover, these changes could induce sequence-independent toxicity mainly observed for high doses of oligonucleotides which include inhibition of the coagulation, activation of the complement cascade and activation of immune response by triggering both innate and adaptive arms (Li and Rana, 2014). These findings demand for additional efforts to improve the pharmacodynamic and pharmacokinetic profiles that, together with advanced delivery system, would increase the therapeutic windows of miRNA-based therapeutics. These and other limitations are extensively discussed elsewhere (van Rooij et al., 2012; Li and Rana, 2014; Zhou et al., 2018) and represent exciting challenges for future research to hopefully move this new class of drug from bench to bedside.

## Concluding Remarks

Overall, miRNAs play an important role in several cardiovascular risk factors and in the pathophysiology of atherosclerosis, cardiac remodeling and MI. These molecules also showed great potential as both biomarkers and therapeutic targets in all of these pathologies, although further research and especially clinical validation is still required. MiRNAs can be a valuable addition to the currently used biomarkers, to further fine-tune diagnostics and come one step closer to the optimal scenario being personalized medicine. In order to use miRNAs in the clinic it is essential that the most promising candidates are further validated in a clinical setting and in different patient cohorts. The use of miRNA targeting as therapeutic strategy is still in its infancy. Pre-clinical studies show very promising results, although most studies are only performed in rodent models so far. It will be very interesting to further study this therapeutic approach in a more translatable setting in order to perform more clinical trials studying the efficacy and safety of these treatments. All in all, it has been extensively demonstrated that miRNAs play an important role in several cardiovascular pathologies and initial studies show great promise for the use of miRNA as biomarkers and even therapeutic targets in the future.

## Author Contributions

LP, EV, and DS contributed to the literature research and writing of the manuscript. EB, MH, and CW contributed to writing of the manuscript and made critical revisions. All authors contributed to the article and approved the submitted version.

## Conflict of Interest

The authors declare that the research was conducted in the absence of any commercial or financial relationships that could be construed as a potential conflict of interest.
